# Investigation of the molecular profile of basal cell carcinoma using whole genome microarrays

**DOI:** 10.1186/1476-4598-5-74

**Published:** 2006-12-15

**Authors:** Lorraine O'Driscoll, Jason McMorrow, Padraig Doolan, Eadaoin McKiernan, Jai Prakash Mehta, Eoin Ryan, Patrick Gammell, Helena Joyce, Norma O'Donovan, Nicholas Walsh, Martin Clynes

**Affiliations:** 1 National Institute for Cellular Biotechnology, Dublin City University, Dublin 9, Ireland; 2 Bons Secours Hospital, Dublin 9 & Blackrock Clinic, Dublin 4, Ireland

## Abstract

**Background:**

Skin cancer accounts for 1/3 of all newly diagnosed cancer. Although seldom fatal, basal cell carcinoma (BCC) is associated with severe disfigurement and morbidity. BCC has a unique interest for researchers, as although it is often locally invasive, it rarely metastasises. This paper, reporting the first whole genome expression microarray analysis of skin cancer, aimed to investigate the molecular profile of BCC in comparison to non-cancerous skin biopsies. RNA from BCC and normal skin specimens was analysed using Affymetrix whole genome microarrays. A Welch t-test was applied to data normalised using dCHIP to identify significant differentially-expressed genes between BCC and normal specimens. Principal component analysis and support vector machine analysis were performed on resulting genelists, Genmapp was used to identify pathways affected, and GOstat aided identification of areas of gene ontology more highly represented on these lists than would be expected by chance.

**Results:**

Following normalisation, specimens clustered into groups of BCC specimens and of normal skin specimens. Of the 54,675 gene transcripts/variants analysed, 3,921 were differentially expressed between BCC and normal skin specimens. Of these, 2,108 were significantly up-regulated and 1,813 were statistically significantly down-regulated in BCCs.

**Conclusion:**

Functional gene sets differentially expressed include those involved in transcription, proliferation, cell motility, apoptosis and metabolism. As expected, members of the Wnt and hedgehog pathways were found to be significantly different between BCC and normal specimens, as were many previously undescribed changes in gene expression between normal and BCC specimens, including *basonuclin2 *and *mrp9*. Quantitative-PCR analysis confirmed our microarray results, identifying novel potential biomarkers for BCC.

## Background

It is estimated that the incidence of cutaneous basal cell carcinoma is increasing worldwide by up to 10% per year [1] and it currently accounts for approximately 80% of all non-melanoma skin cancer – with highest rates in elderly men and increasing incidence in young women [2]. Several sub-types of BCC have been identified. These include nodular-ulcerated BCC (the most frequently occurring type; often with ulceration ("rodent ulcer")); superficial BCC (often multiple); sclerosing BCC (cancer cells surrounded by dense fibrosis and so resemble scars; highest recurrence rate of BCCs after treatment); cystic BCC (uncommon; tumour undergoes central degradation to form a cystic lesion); linear BCC (recently recognised clinical entity with increased risk for aggressive histopathology); and micronodular BCC (small tumour nests; often with subclinical growth). Although BCC only occasionally (0.003–0.55% of cases [3-5]), results in metastasis and is seldom fatal, BCC is often locally invasive with destructive growth and may be associated with severe disfigurement and morbidity as a result of local tissue destruction or due to necessary surgery. Furthermore, people with BCC are at higher risks of developing further BCCs and other malignancies, including squamous cell carcinomas, malignant melanomas, and possibly also non-cutaneous malignancies [1].

Current selection of best treatment for BCC is based on accurate diagnosis and sub-classification of these cancers, mainly on histomorphology/pathology of H&E stained sections [6]. Features associated with recurrence and metastasis are also considered – including tumour diameter >2 cm, location in the central part of the face or ear, present for long duration, incomplete excision, aggressive growth pattern (based on histology) and perinuclear or perivascular involvement [2]. Genes reported to be associated with susceptibility to BCC include CYP2D6, GST-T1, vitamin D receptor, and TNF; with UVB irradiaton known to cause mutations in the p53 tumour suppressor gene, leading to the development of this cancer [1].

With the exception of a single study of BCCs using a small cDNA microarray – representing 1,718 genes [7] – investigations aimed at identifying BCC biomarkers and understanding the molecular events involved in this disease have, in general, been limited to one-at-a-time studies, built on chance analyses of proteins or mRNAs. Examples of such protein analysis have identified CD10 [8,9], p63 [10], low expression levels of CD44 [11] to be associated with the presence of BCC, generally with absence of ICAM-1 and LFA-3 adhesion molecule expression [12] and with Ki67 expression levels differing between BCCs that recur, compared to those that do not recur [13]. RT-PCR analysis has indicated *1,25-dihydroxyvitamin D(3) receptor *mRNA levels to be increased in BCCs compared to normal skin [14], while qPCR quantification of *gli1 *transcripts has been found to discriminate BCC (and trichoepithelioma) from other skin cancers [15].

While such studies have indicated the importance and relevance of gene expression analyses in BCC, the number of gene products simultaneously analysed have been very limited. In order to increase our understanding of the molecular events involved in the development/expression of BCC, here we report our findings from whole genome microarray analysis of BCC and normal skin specimens.

## Results

### Quality Control of Microarray Data

As indicated in Table 1, quality control (Q.C.) analysis of all 25 microarray data sets (from 20 BCC and 5 normal skin specimens) indicated an average percentage present call of 42.68% (+/- 5.79 SD). This would be of the order expected for high quality RNA from cell lines from many origins (Affymetrix Inc. "Genechip^® ^Expression Analysis Data Analysis Fundamentals" [16], indicating that these results are acceptable for further analysis. Again, based on cell line Q.C. parameters, the accepted background levels are <100, while 52.31 +/- 4.08 was found in this study; acceptable noise levels are <3, here we report 1.67 +/- 0.4; and the acceptable scaling factor is <3 fold between data sets being compared. This was generally, but not always, achieved. The acceptable 3'/M ratio of <3 was achieved in 18/25 cases.

**Table 1:** Q.C. Analysis of Microarray Results

**Specimen I.D.**	**Present Call (%)**	**Background**	**Noise**	**Scaling Factor**	**3'/M Ratio GAPDH**
BCC26	49.9	52.6	1.82	0.94	14.56
JT2	50.2	63.6	2.05	0.795	1.41
JT3	49.9	53.85	1.74	1.037	1.27
BCC4	48.8	55.72	1.87	0.868	1.82
JT6	41.8	50.33	1.56	2.431	2.46
JT11	39	48.93	1.58	3.173	2.17
JT8	43	46.94	1.48	2.463	1.95
JT9	46.5	53.85	1.71	1.434	1.66
T16	43.4	51.87	1.62	1.896	3.25
JT12	38.1	53.09	1.7	2.709	2.86
JT13	47.1	54.46	1.67	1.374	1.67
JT4	46.5	55.73	1.77	1.18	1.66
JT5	45.8	53.86	1.75	1.453	1.86
T24	38.5	57.3	1.83	2.905	3.0
T25	34.1	56.57	1.77	3.988	3.1
T28	34.3	56.32	1.79	3.771	3.6
JT7	39.6	48.25	1.56	3.098	2.52
T11	43.3	50.10	1.61	2.416	2.1
T19	32.2	54.24	1.7	4.539	3.1
T22	29.1	49.14	1.59	6.639	5.86
N1	46.6	46.38	1.46	1.571	1.67
N2	44.0	47.85	1.56	1.565	1.8
N3	42.5	50.84	1.63	2.105	2.46
N5	46.7	48.96	1.54	1.417	1.85
N6	46.2	46.92	1.44	1.821	2.39

*Note*: Acceptable Q.C. cut-offs, based on high quality cell line RNA analysis, for background is <100; noise <3; scaling factor <3 fold between specimens being compared; 3'/M ratio <3 (Affymetrix Inc. "Genechip^® ^Expression Analysis Data Analysis Fundamentals" [16]).

### Data Analysis

**Figure 1:**
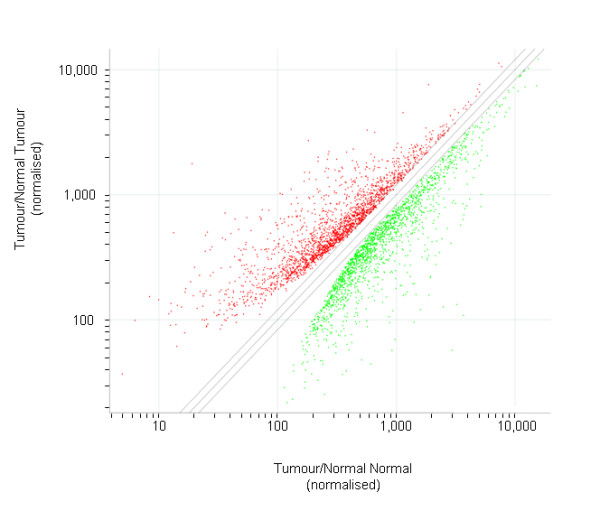
Scatter Plot of the 3,921 gene transcripts identified as significantly differentially expressed (by ≥1.2 fold; ≥100 difference in expression intensity; P < 0.05) between BCC and normal skin specimens. Transcripts significantly up-regulated are shown in red; those down-regulated are shown as green.

Approximately 7% (3,921/54,675) of the probe sets representing transcripts on the microarray were significantly differentially expressed between BCC and normal skin specimens (Tables 2 &3; [see Additional Files 1 &2] for further information). The scatter plot of data differentially expressed ≥1.2 fold (Fig. 1) indicates an even, normal distribution of data. As shown in Table 4, of the 2,108 up-regulated by ≥1.2 fold, genes involved in many crucial aspects of cellular biology, including metabolism, transcription, cell cycle regulation, cell adhesion, cell migration, cell proliferation and cell motility were amongst the largest groups of genes affected, while oxidative phosphorylation, lipid metabolism, translation, and apoptosis were among the main categories down-regulated ≥1.2 fold in BCCs compared to normal skin (Table 5). Of the 748 probesets representing transcripts up-regulated by ≥2 fold, approximately 11 were described as cloned cDNAs, 122 were ESTs, 6 were described as hypothetical genes, and 46 encoded hypothetical proteins. Of the 484 transcripts showing ≥2 fold down-regulation, twenty-five represented hypothetical proteins, 11 cloned cDNAs/RIKENS and 49 ESTs.

**Table 2:** Gene Transcripts significantly up-regulated in BCC compared to normal skin specimens (≥1.2 fold; ≥100; p < 0.05).

**probe set**	**gene**	**Accession**	**fold change**	**Difference**	**P value**
204697_s_at	chromogranin A (parathyroid secretory protein 1)	NM_001275	130.34	2497.11	0.000001
224590_at	X (inactive)-specific transcript	BE644917	69.08	427.18	0.001884
214218_s_at	X (inactive)-specific transcript	AV699347	62.79	621.31	0.00072
242964_at	gb:AI421677/DB_XREF=gi:4267608/DB_XREF=tf54a03.x1/CLONE	AI421677	55.54	730.97	0.000005
224588_at	X (inactive)-specific transcript	AA167449	49.82	2086.08	0.000552
204913_s_at	SRY (sex determining region Y)-box 11	AI360875	27.92	228.19	0.000224
1560652_at	gb:AL832136.1/DB_XREF=gi:21732679/TID=Hs2.407141.1/CNT=4	AL832136	26.21	638.38	0.000015
236029_at	FAT tumor suppressor homolog 3 (Drosophila)	AI283093	24.03	916.82	0.000015
214913_at	a disintegrin-like and metalloprotease (reprolysin type)	AB002364	23.97	605.9	0.000002
220345_at	leucine rich repeat transmembrane neuronal 4	NM_024993	22.24	270.15	0.0061
233622_x_at	Transcribed locus, weakly similar to XP_375099.1 hypothetical protein	AL162077	21.31	105.45	0.005425
230863_at	gb:R73030/DB_XREF=gi:847062/DB_XREF=yj94c11.s1/CLONE=	R73030	20.05	292.94	0.029359
204915_s_at	SRY (sex determining region Y)-box 11	AB028641	19.64	524.92	0.000059
204424_s_at	LIM domain only 3 (rhombotin-like 2)	AL050152	19.19	1287.59	0.003562
236407_at	gb:R73518/DB_XREF=gi:847550/DB_XREF=yj93h12.s1/CLONE=	R73518	18.1	355.25	0.000003
208025_s_at	high mobility group AT-hook 2///high mobility group AT-hook 2	NM_003483	17.76	506.18	0.000173
215311_at	MRNA full length insert cDNA clone EUROIMAGE 21920	AL109696	17.1	448.46	0.000013
227671_at	X (inactive)-specific transcript	AV646597	16.98	383.7	0.003059
218638_s_at	spondin 2, extracellular matrix protein	NM_012445	16.91	2992.93	0
208212_s_at	anaplastic lymphoma kinase (Ki-1)	NM_004304	16.76	835.41	0.000003
226346_at	gb:AA527151/DB_XREF=gi:2269220/DB_XREF=ni07b08.s1/CLONE	AA527151	15.34	534.55	0
204914_s_at	SRY (sex determining region Y)-box 11	AW157202	15.1	310.64	0.000124
215443_at	thyroid stimulating hormone receptor	BE740743	14.95	239.99	0.00019
240460_at	gb:AI190616/DB_XREF=gi:3741825/DB_XREF=qd38e02.x1/CLONE	AI190616	14.91	168.09	0.000564
1562107_at	hypothetical protein MGC14738	BC007100	14.73	284.47	0.000263
213960_at	CDNA FLJ37610 fis, clone BRCOC2011398	T87225	14.18	534.88	0.000001
1565936_a_at	LIM domain only 3 (rhombotin-like 2)	T24091	13.94	162.48	0.001811
229523_at	gb:N66694/DB_XREF=gi:1218819/DB_XREF=yy71g08.s1/CLONE=	N66694	13.82	614.51	0.000001
210055_at	thyroid stimulating hormone receptor	BE045816	13.81	181.7	0.000595
224646_x_at	gb:BF569051/DB_XREF=gi:11642431/DB_XREF=602184410T1/	BF569051	13.81	1024.01	0.000261
207468_s_at	secreted frizzled-related protein 5	NM_003015	13.54	437.41	0.001339
220518_at	target of Nesh-SH3	NM_024801	13.44	448.75	0.001398
224997_x_at	H19, imprinted maternally expressed untranslated mRNA	AL575306	13.39	1131.86	0.000494
222940_at	sulfotransferase family 1E, estrogen-preferring, member 1	U55764	13.29	560.15	0.000001
235795_at	gb:AW088232/DB_XREF=gi:6044037/DB_XREF=xc99c09.x1	AW088232	13.26	199.49	0.000451
220090_at	chromosome 1 open reading frame 10	NM_016190	12.79	736.69	0.00414
238584_at	IQ motif containing with AAA domain	W52934	12.68	222.06	0.001169
203878_s_at	matrix metalloproteinase 11 (stromelysin 3)	NM_005940	12.23	685.83	0.000417
1557215_at	Transcribed locus, weakly similar to XP_375935.1 hypothetical protein	AK056212	11.92	262.54	0.003503
210292_s_at	protocadherin 11 Y-linked///protocadherin 11 X-linked	AF332218	11.83	211.94	0.000324
1558964_at	FAT tumor suppressor homolog 3 (Drosophila)	AA334950	11.22	280.34	0.000123
214451_at	transcription factor AP-2 beta (activating enhancer binding protein 2	NM_003221	10.67	2196.21	0
209816_at	patched homolog (Drosophila)	AL044175	10.59	245.97	0.001175
241617_x_at	gb:BE961949/DB_XREF=gi:11764352/DB_XREF=601655369R1	BE961949	10.43	1011	0.000972
209815_at	Patched homolog (Drosophila)	BG054916	10.34	2145.95	0.000007
230496_at	Hypothetical protein FLJ25477	BE046923	10.34	131.12	0.000467
205372_at	pleiomorphic adenoma gene 1	NM_002655	10.1	1069.14	0
229942_at	gb:AW024890/DB_XREF=gi:5878420/DB_XREF=wu92c11.x1	AW024890	9.98	1649.01	0
214297_at	gb:BE857703/DB_XREF=gi:10371993/DB_XREF=7g46a02.x1	BE857703	9.86	819.59	0.000003
229669_at	Hypothetical protein LOC339260	AA166965	9.83	237.59	0.001318

Top 50 transcripts – based on fold difference are shown (complete list is supplied as supplementary material).

**Table 3:** Gene Transcripts significantly down-regulated in BCC compared to normal skin specimens (≥1.2 fold; ≥100; p < 0.05).

**probe set**	**gene**	**Accession**	**fold change**	**Difference**	**P value**
239929_at	hypothetical protein FLJ32569	AA918425	-28.24	-3433.29	0.014118
208962_s_at	fatty acid desaturase 1	BE540552	-20.2	-3188.7	0.045399
229476_s_at	thyroid hormone responsive (SPOT14 homolog, rat)	AW272342	-17.15	-4660.08	0.034919
207275_s_at	acyl-CoA synthetase long-chain family member 1	NM_001995	-15.11	-1865.7	0.039975
206799_at	secretoglobin, family 1D, member 2	NM_006551	-14.97	-1291.31	0.042413
234513_at	elongation of very long chain fatty acids (FEN1/Elo2, SUR4/Elo3, yeast)-like	AF292387	-13.28	-670.81	0.022544
221561_at	sterol O-acyltransferase (acyl-Coenzyme A: cholesterol acyltransferase) 1	L21934	-12.23	-998.57	0.008422
206714_at	arachidonate 15-lipoxygenase, second type	NM_001141	-11.45	-3891.76	0.045644
214240_at	galanin	AL556409	-11.12	-1590.25	0.015802
244661_at	gb:AA946876/DB_XREF=gi:3110271/DB_XREF=oq53c11.s1/CLONE=	AA946876	-10.56	-399.74	0.017168
244780_at	sphingosine-1-phosphate phosphotase 2	AI800110	-9.79	-351.6	0.024348
201625_s_at	insulin induced gene 1	BE300521	-9.72	-1591.34	0.030044
231810_at	BRI3 binding protein	BG106919	-8.9	-877.2	0.021503
1565162_s_at	microsomal glutathione S-transferase 1	D16947	-8.6	-1071.37	0.030259
238121_at	Transcribed locus, weakly similar to XP_341569.1 similar to ORF4	AI473796	-8.29	-445.99	0.042733
211056_s_at	steroid-5-alpha-reductase, alpha polypeptide 1 (3-oxo-5 alpha-steroid delta	BC006373	-7.88	-1432.56	0.032165
229957_at	Branched chain keto acid dehydrogenase E1, alpha polypeptide	BF446281	-7.63	-1214.3	0.025272
204675_at	steroid-5-alpha-reductase, alpha polypeptide 1 (3-oxo-5 alpha-steroid delta	NM_001047	-7.35	-2832.7	0.027894
233030_at	adiponutrin	AK025665	-7.35	-1220.35	0.031698
231736_x_at	microsomal glutathione S-transferase 1	NM_020300	-7.28	-2497.22	0.031804
205029_s_at	fatty acid binding protein 7, brain	NM_001446	-7.24	-524.52	0.021739
201627_s_at	insulin induced gene 1	NM_005542	-7.23	-627.89	0.049971
209522_s_at	carnitine acetyltransferase	BC000723	-7.23	-2039.08	0.020824
226064_s_at	diacylglycerol O-acyltransferase homolog 2 (mouse)	AW469523	-7.1	-2291.54	0.032748
231156_at	gb:AW242782/DB_XREF=gi:6576459/DB_XREF=xm89g06.x1/CLONE	AW242782	-7.09	-339.26	0.045195
223184_s_at	1-acylglycerol-3-phosphate O-acyltransferase 3	BC004219	-7.01	-1374.94	0.045149
232428_at	monoacylglycerol O-acyltransferase 2	AK000245	-6.97	-182.68	0.038707
1558846_at	Pancreatic lipase-related protein 3	AL833418	-6.71	-2007.72	0.044402
205030_at	fatty acid binding protein 7, brain	NM_001446	-6.41	-1864.48	0.021392
205843_x_at	carnitine acetyltransferase	NM_000755	-6.41	-1166.98	0.020573
224435_at	chromosome 10 open reading frame 58///chromosome 10 open reading	BC005871	-6.37	-1905.14	0.007356
1560507_at	Diacylglycerol O-acyltransferase 2-like 3	BC039181	-6.28	-441.54	0.028259
215726_s_at	cytochrome b-5	M22976	-5.95	-3458.82	0.02416
220431_at	DESC1 protein	NM_014058	-5.94	-112.38	0.029175
225716_at	gb:AI357639/DB_XREF=gi:4109260/DB_XREF=qy15b05.x1/CLONE=	AI357639	-5.94	-1718.34	0.01598
204388_s_at	monoamine oxidase A	NM_000240	-5.89	-362.75	0.037779
45288_at	abhydrolase domain containing 6	AA209239	-5.83	-698.25	0.020361
218804_at	transmembrane protein 16A	NM_018043	-5.75	-234.79	0.045057
228479_at	gb:AI094180/DB_XREF=gi:3433156/DB_XREF=qa29b09.s1/CLONE=	AI094180	-5.7	-1191.04	0.029615
227804_at	hypothetical protein BC014072	BE328850	-5.62	-363.64	0.043913
234312_s_at	acetyl-Coenzyme A synthetase 2 (ADP forming)	AK000162	-5.54	-1297.68	0.044265
1562528_at	RAR-related orphan receptor A	BC040965	-5.48	-120.02	0.025055
208964_s_at	fatty acid desaturase 1	AL512760	-5.46	-3305.87	0.025757
213693_s_at	gb:AI610869/DB_XREF=gi:4620036/DB_XREF=tp21e08.x1/CLONE=	AI610869	-5.43	-1427.55	0.023253
237507_at	keratin 6 irs3	AI333069	-5.43	-271.6	0.008082
204389_at	monoamine oxidase A	NM_000240	-5.4	-180.28	0.0359
214598_at	claudin 8	AL049977	-5.27	-370.2	0.01827
201963_at	acyl-CoA synthetase long-chain family member 1	NM_021122	-5.19	-4459.79	0.010049
218434_s_at	acetoacetyl-CoA synthetase	NM_023928	-5.17	-1842.1	0.004574
221552_at	abhydrolase domain containing 6	BC001698	-5.06	-402.38	0.024809

Top 50 transcripts – based on fold difference are shown (complete list is supplied as supplementary material).

**Table 4:** GOstat analysis of biological processes.

**GO Number**	**Observed Transcripts**	**Total Transcripts**	**GO Category**	**P value**
GO:0019222	293	3309	regulation of metabolism	1.24E-33
GO:0006351	264	2884	transcription, DNA-dependent	5.41E-33
GO:0006355	258	2809	regulation of transcription, DNA-dependent	1.61E-32
GO:0006357	55	280	regulation of transcription from RNA polymerase II promoter	5.40E-31
GO:0008134	57	346	transcription factor binding	6.86E-24
GO:0016563	45	250	transcriptional activator activity	9.36E-22
GO:0003677	240	2906	DNA binding	1.54E-21
GO:0003712	44	276	transcription cofactor activity	3.54E-17
GO:0007049	89	807	cell cycle	9.89E-17
GO:0016043	163	1986	cell organization and biogenesis	8.78E-14
GO:0003713	30	171	transcription coactivator activity	1.08E-13
GO:0008283	65	560	cell proliferation	1.17E-13
GO:0051726	61	522	regulation of cell cycle	5.62E-13
GO:0000074	60	518	regulation of progression through cell cycle	1.51E-12
GO:0005578	48	377	extracellular matrix	3.00E-12
GO:0007155	77	874	cell adhesion	5.65E-08
GO:0008380	24	173	RNA splicing	3.60E-07
GO:0016477	18	112	cell migration	5.56E-07
GO:0016055	19	128	Wnt receptor signaling pathway	2.09E-06
GO:0008219	60	675	cell death	2.29E-06
GO:0007167	25	207	enzyme linked receptor protein signaling pathway	1.13E-05
GO:0030154	50	550	cell differentiation	1.13E-05
GO:0005604	13	62	basement membrane	7.37E-05
GO:0051301	22	190	cell division	0.000156
GO:0042981	38	418	regulation of apoptosis	0.000269
GO:0000904	11	66	cellular morphogenesis during differentiation	0.00262
GO:0043123	13	91	positive regulation of I-kappaB kinase/NF-kappaB cascade	0.00344
GO:0005581	11	73	collagen	0.00597
GO:0030198	8	43	extracellular matrix organization and biogenesis	0.00788
GO:0007154	247	4475	cell communication	0.0122
GO:0005583	4	11	fibrillar collagen	0.0125
GO:0008286	5	20	insulin receptor signaling pathway	0.0182
GO:0016049	17	174	cell growth	0.019
GO:0008361	17	174	regulation of cell size	0.019
GO:0005610	2	2	laminin-5	0.0205
GO:0008083	20	221	growth factor activity	0.0232
GO:0005588	2	3	collagen type V	0.0477

Biological processes that are significantly enriched in our set of 2,108 transcripts found to be significantly up-regulated in BCC compared to normal skin specimens. Shown here is a sub-set of 37 representative significant GO annotations.

**Table 5:** GOstat analysis of biological processes.

**GO Number**	**Observed Transcripts**	**Total Transcripts**	**GO Category**	**P value**
GO:0006119	36	138	oxidative phosphorylation	2.77E-34
GO:0006091	120	1003	generation of precursor metabolites and energy	3.75E-33
GO:0016491	123	1170	oxidoreductase activity	7.47E-26
GO:0006732	44	269	coenzyme metabolism	5.71E-21
GO:0006629	86	765	lipid metabolism	1.17E-20
GO:0003954	24	62	NADH dehydrogenase activity	8.07E-16
GO:0043037	40	280	translation	4.26E-15
GO:0016651	27	99	oxidoreductase activity, acting on NADH or NADPH	1.63E-13
GO:0016126	16	31	sterol biosynthesis	5.82E-13
GO:0008135	29	185	translation factor activity, nucleic acid binding	8.99E-13
GO:0045045	30	217	secretory pathway	1.31E-10
GO:0006099	14	45	tricarboxylic acid cycle	5.49E-08
GO:0046356	14	45	acetyl-CoA catabolism	5.49E-08
GO:0006511	25	201	ubiquitin-dependent protein catabolism	2.83E-07
GO:0006413	17	84	translational initiation	1.20E-06
GO:0048193	19	108	Golgi vesicle transport	2.00E-06
GO:0006412	87	1228	protein biosynthesis	5.29E-06
GO:0008289	35	380	lipid binding	2.46E-05
GO:0006445	15	92	regulation of translation	7.85E-05
GO:0006888	13	72	ER to Golgi vesicle-mediated transport	9.90E-05
GO:0006915	49	644	apoptosis	0.000214
GO:0008415	20	190	acyltransferase activity	0.000313
GO:0006944	7	32	membrane fusion	0.00289
GO:0016281	4	9	eukaryotic translation initiation factor 4F complex	0.00313
GO:0006984	4	9	ER-nuclear signaling pathway	0.00313
GO:0044242	7	33	cellular lipid catabolism	0.00349
GO:0030503	3	5	regulation of cell redox homeostasis	0.00611
GO:0007050	11	85	cell cycle arrest	0.00717
GO:0016282	5	27	eukaryotic 43S preinitiation complex	0.0303

Biological processes that are significantly enriched in our set of 1,813 transcripts found to be significantly down-regulated in BCC compared to normal skin specimens. Shown here is a sub-set of 29 representative significant GO annotations.

**Figure 2:**
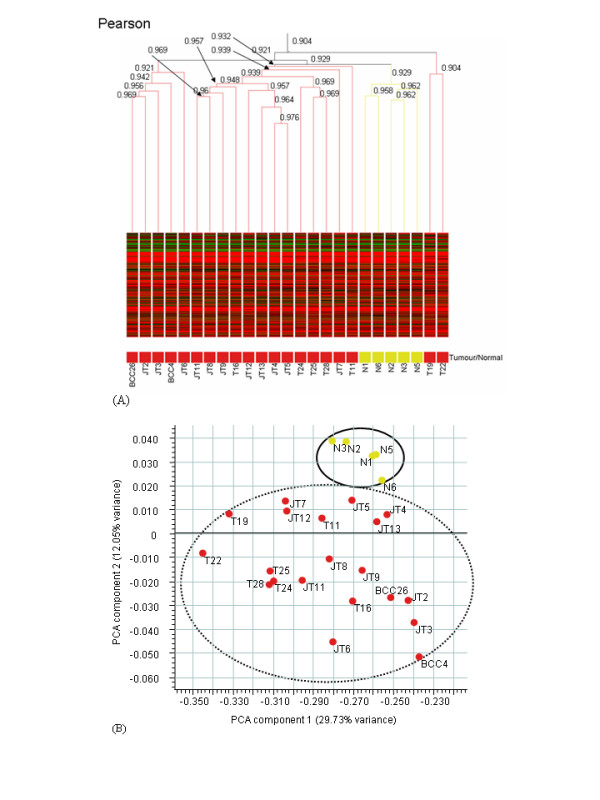
**(A) **Condition tree distribution of the 20 BCC (*red*) and 5 normal skin (*yellow*) specimens (following dCHIP normalisation, similarity measure, Pearson's correlation, clustering algorithm, average linkage). Expression values ≥100 are indicated in red; ≥50 to <100 are indicated in black, and 0 to <50 are indicated in green. **(B) **Two-dimensional principal component analysis (PCA) plot where red dots represent BCC specimens and yellow dots represent normal skin specimens indicating that while the normal specimens form a loose cluster (*solid line oval*), the BCC specimens are more "scattered" and varied (*broken line oval*). The first principal component expression value is 29.73%; the second component expression value is 12.05%.

In order to identify specimen similarity/diversity in our group of 25 skin specimens, condition tree clustering (using Pearson's correlation coefficient as similarity metric) and principal component analysis (PCA) were performed using GeneSpring software. As indicated in Fig. 2(A), while Pearson's correlation coefficient does not suggest a significant difference (*i.e*. no values <0.9) between the 25 specimens analysed, the 5 normal specimens form a discrete cluster in relation to the BCC specimens. (A similar clustering pattern was obtained when Spearman's correlation coefficient was investigated). Two of the BCC specimens, T19 and T22, are apparently different (but not significantly different) to the other 18 BCCs. While the 3'/M ratios were greater than expected (at least in comparison to cell line data) for these specimens, this was also the case for BCC26, T16, T24, T25 and T28, which did not group with T19 and T22. The higher scaling factor resulting from analysis of T19 and T22, compared to all other specimens, may be responsible for/contribute to their apparently somewhat different behaviour as represented on the condition tree. For the purpose of investigating the effects of T19 and T22 on the overall dataset, a re-analysis was performed excluding data relating to those specimens. This resulted in a reduction from 3,921 (*i.e*. 7.17%) to 3,865 (7.06%) of significantly differentially expressed transcripts between BCC and normal specimens, indicating that the vast majority of these transcripts are unbiased by the slightly different behaviour of T19 and T22 compared to all other BCCs (as described above).

To further identify the relatedness of the BCC samples to each other, we performed principal components analysis [17] on the entire data set. PCA was carried out on log-transformed data, using mean centering and scaling. As can be seen in Fig. 2(B), while our PCA analysis has divided the specimens into two groups, the results indicate that the BCC sub-group is much more varied than the normal skin group and that the BCC and normal skin specimens do not differ greatly.

Support Vector Machine analysis [18] is a machine learning classification approach which is suitable for application to the dimensionality of microarray data. It operates by examining the expression information of a set of data points whose classification is known (referred to as the "training set"), from which a defined number of classification predictor genes are identified. This predictor genelist can then be applied to a separate set of genes which are known not to be members of the functional class (referred to as the "test set"). The predictor genelist is user-defined, so it is more beneficial to the user for validation if a smaller number of genes comprise the predictor list.

For the purposes of this study, we carried out SVM analysis repeatedly on the tumour vs. normal dataset, in order to identify the lowest number of probesets that discriminated 100% of the time between the two classifications. The gene selection method used was Fisher's Exact Test, the kernel function was Polynomial Dot Product (Order 1) with zero Diagonal Scaling Factor. A minimum number of six identifiers, shown in Table 6, were required for 100% classification of every specimen as either tumour or normal in origin. Attempts to further prioritise these genes resulted in a decrease in the efficiency of the classification, as further analysis aimed at classifying the specimens using less than six transcripts resulted in the mis-classification of normal specimen N5 as a BCC specimen.

**Table 6:** Transcripts identified using Support Vector Machine as suitable, as a group, for 100% classification of BCC from normal skin.

**I.D.**	**Gene Transcript**
1553718_at	zinc finger protein 548
201413_at	hydroxysteroid (17-beta) dehydrogenase 4
225677_at	B-cell receptor-associated protein 29
223184_s_at	1-acylglycerol-3-phosphate O-acyltransferase 3
203878_s_at	matrix metalloproteinase 11 (stromelysin 3)
225716_at	Full-length cDNA clone CS0DK008YI09 of HeLa cells Cot 25-normalized of Homo sapiens (human)

### qPCR Validation of Microarray Data

Quantitative-PCR (qPCR) analysis for 10 potential endogenous controls was performed on a random selection of four BCC and four normal skin specimens, to select a suitable endogenous control(s) amplifiable in all specimens and where levels of expression did not differ greatly between specimens being analysed. All 10 transcripts studied were found to be expressed in all specimens analysed and, as indicated in Fig. 3 [Additional File 3], expression levels and standard deviation results suggested that the 10 transcripts are likely to be of similar suitability as endogenous controls. Two controls, β-actin and GAPDH, were subsequently selected for amplification in all specimens, in parallel with a selection of 5 target transcripts of interest. The relative expression levels of *PTCH1*, *gli2, Frizzled D2, basonuclin 2, and chromagranin A *were analysed by qPCR. As indicated in Table 7, while the fold differences detected by microarray and qPCR methods differed to some extent, the trend (*i.e*. absent/low expression in normal skin and induced/increased expression in BCCs) was always found.

**Table 7:** Validation of microarray data by qPCR

**Transcript**	**I.D.**	**Microarray^1 ^Mean Expression Levels in Normal Skin**	**Microarray^1 ^Mean Expression Levels in BCC**	**Microarray (*fold*)^2^**	***qPCR*^3 ^Mean Expression Levels in Normal Skin**	***qPCR*^3 ^Mean Expression Levels in BCC**	***qPCR *(*fold*)^2^**
*chromagranin A*	NM_001275	19.31	2516.41	130.34	0.000	1.083	P
*gli2*	NM_030379	23.51	173.73	7.39	0.000	1.662	P
*PTCH1*	BG054916	229.86	2375.81	10.34	0.025	1.455	58.2
*basonuclin 2*	NM_017637	204.42	1827.2	8.94	0.077	1.034	13.4
*frizzled 2*	L37882	54.93	490.8	8.94	0.047	6.505	138.4

*Note*: ^1 ^= Expression levels, Affymetrix arbitrary units; ^2 ^= fold increased expression in BCC compared to normal; ^3 ^= following normalisation of data on mean of β-actin + GAPDH expression and calibrating to a pooled skin specimen control (as described in Materials and Methods); P = induced in BCC, while undetected in normal skin

## Discussion

Basal cell carcinoma (BCC), the most common skin cancer in humans, is locally aggressive and relentlessly invasive, but generally does not metastasise [5,19]. Despite this, studies aimed at investigating the molecular mechanisms associated with – possibly responsible for – BCCs, have been very limited. In 2005, Howell *et al*. [7] reported findings from their analysis of 1,718 transcripts in BCC specimens, using cDNA microarrays. While this study produced very interesting results, as explained by the authors, numbers of transcripts potentially important in BCC – such as *PTCH1 *and *SMO *– were not represented on their microarray; limiting their study considerably. Here we have successfully analysed gene expression of BCCs, compared to normal skin, using whole genome microarrays and following extensive analysis of our data, in addition to confirming previous findings, we have identified a number of novel potential biomarkers/therapeutic targets for this disease.

Comparison of our results with those generated by Howell *et al*. [7] indicated a relatively high level of agreement between these two studies. Many of the transcripts identified by Howell *et al*. as up-regulated in BCC compared to normal skin were also found to be up-regulated in BCC in our study. Examples of these include collagens (*type V, alpha 1 & alpha 2; type IV alpha 1 & 2; type VII alpha 1*), *topoisomerase II*α, *tumour-associated calcium signal transducer 1*, *profilin 2*, *calretinin*, *syndecan 2*, and *v-myc*. Similarly, a high concordance between these two studies was found for transcripts down-regulated in BCCs compared to normal specimens; examples of which include *cystatin B*, *acetyl-Coenzyme acyltransferase 1, 3-hydroxy-3-methylglutaryl-Coenzyme A reductase*, *glutaredoxin*, *amyloid *β *(A4) precursor-like protein*, and *cytochrome b-5*. However, in the case of a limited number of differentially expressed transcripts, the direction of change in expression in BCC compared to normal skin was not in agreement. Examples include *ADP-ribosylation factor 3 *(down-regulated by 1.67 fold in our study [expression values 941.51 vs. 563.45], but up-regulation reported by Howell *et al*. [7] and *glia maturation factor *β (1.42 up-regulated in our analysis [expression values 969.02 vs. 1,377.23], but reported by Howell *et al*. as down-regulated). While the reasons for these limited number of discrepencies is unknown, it may be due to different splice variants of these transcripts being detected by cDNA compared to oligo microarrays. It is worth noting that the results that differed between our study and that of Howell *et al*. were generally transcripts that we found to be <2 fold differentially expressed between BCC and normal skin. Unfortunately, as fold changes observed by Howell *et al*. [7] were not reported in their manuscript and information is not publicly available on transcripts that were present on their microarray, but were not significantly changed, further comparisons between these studies cannot be performed.

The development of BCC is known to be associated with dys-regulation of the hedgehog and Wnt pathways [2,20]. Lack of expression and/or suppressed activation of patched homologue 1 (PTCH1), a tumour suppressor gene that forms part of the hedgehog signaling network [21], has been reported to be fundamental to the development of BCC [22]. Disruption of this tumour suppressor gene results in up-regulated cell proliferation [6]. The accepted mechanism of PTCH1's action is via its binding to another transmembrane molecule, smoothed (SMO), thus suppressing intracellular signaling. Following binding of sonic hedgehog (shh) to PTCH1, this suppressor activity is, however, quenched, resulting in uninterrupted signal transduction by SMO, *via *GLI transcription factors, and subsequent constitutive activation of target genes, including members of the Wnt pathway [23] and PTCH1, itself [21]. From our analysis of BCC compared to normal skin tissue, while we found no significant changes in *shh *expression levels, we report an approximately 11 fold increased expression of *PTCH1 *[expression values 25.65 vs. 271.62], and also increased expression of *gli2 *(7.39 fold; P = 0.00009) [expression values 23.52 vs. 173.73], respectively. The induced/increased expression of *PTCH1 *and *gli2 *found by microarray analysis was confirmed by qPCR analysis of all specimens (see Table 7). It is important to note that the likely reason for the difference in fold expression detected by microarray and qPCR methods is due to their differing baseline sensitivity. Low expression levels detected by microarrays – *e.g*. *gli2 *in normal skin – resulting in large fold differences are considered as induction from absent in normal skin compared to present in BCC specimens, when analysed by qPCR. However, although *PTCH1 *mRNA levels have previously been reported as enhanced in nodular BCC but undetectable in superficial BCC [24], here we report *PTCH1 *to be detectable in both of these histological types of BCC, with no significant difference in their respective expression values (t-test: p = 0.637).

While the increased expression of *gli2 *detected in BCCs compared to normal skin may be expected and associated with the development/presence of BCC, the lack of tumour suppressor activity by PTCH1 – despite its increased mRNA levels – may be due to lack of expression of its corresponding protein and/or lack of binding to *SMO *(levels of which were not significantly different between BCC and normal skin). As PTCH1 may shuttle between the cell membrane and endocytotic vesicles in response to active hedgehog ligand, it is obvious that not only its mRNA expression, but also its protein expression (at the relevant location, binding of SMO, *etc*.) is necessary to exert its tumour suppressor activity [21]. Furthermore, as loss-of-function mutations of PTCH1 have been identified in 30–40% of sporadic cases of BCC, it may be that the mRNA over-expressed in the BCCs is not coding for a functional protein.

Wnt signaling may be able to regulate a number of the aspects of the biology of tumour cells and thus contribute in several ways to the tumour phenotypes. The strongest link is to the control of proliferation. Knockouts of Wnt signal transduction components, including Wnt5A, can result in proliferative failure [25] while up-regulation of *Wnt5A *mRNA expression been associated with a range of cancer types, including breast, lung, prostate and malignant melanomas [26,27]. In our study, the involvement of Wnt signaling pathway in BCC is suggested by the significantly increased expression of a number of Wnt family members. These include *Wnt5A *(3.35 fold; P = 0.00003) [expression values 403.73 vs. 1,353.71], – in agreement with a study by Saldanha *et al*. [19] where Wnt5A levels were increased in BCCs compared to surrounding skin – and *Wnt6 *(4.86 fold; P = 0.00002) [expression values 59.7 vs. 290.26]. Increased levels of Wnt ligand binding receptors, *Frizzled D2 *(8.94 fold; P = 0.000033) [expression values 54.93 vs. 490.8], *D7 *(2.31 fold; P = 0.000085) [expression values 276.23 vs. 638.34], and *D8 *(5.89 fold; P = 0.000055) [expression values 44.44 vs. 261.74], and decreased levels of *D4 *(-2.78 fold; P = 0.02) [expression values 598.41 vs. 215.2], were also found. The increased expression of *Frizzled D2 *in BCC compared to normal skin was confirmed by qPCR.

In the "canonical" Wnt signaling pathway, secreted ligands bind to Frizzled receptors and regulate the stability of β-catenin. (Given the large number of mammalian Frizzleds and Wnts, considerable ligand-receptor specificity might be expected; however, redundancy of function seems to be the rule [25]). The subsequent accumulation of β-catenin – the central player in the Wnt pathway [28] – in the nucleus, results in its participation in transcriptionally active complexes with members of the LEF/TCF family of transcription factors [29]. While we did not find levels of β-catenin to be significantly different between BCC and normal skin, decreased (-2.1 fold; P = 0.006) [expression values 2,090.09 vs. 992.89], levels of *CTNNBIP1*, an inhibitor of β-catenin and TCF-4 (ICAT) which would normally prevent β-catenin binding to LEF transcription factors [30-32] and increased levels of *LEF1 *transcripts (3.42 fold; P = 0.000001) [expression values 513.06 vs. 1,752.32], were found. Levels of *Jun *(2.34 fold; P = 0.00006) [expression values 875.92 vs. 2,052.07], another transcription factor involved in the Wnt pathway [33], were also found to be increased in BCCs compared to normal skin. Differential expression of other transcription factors associated with cancer has also been found in this study. These include *CHES1 *(*checkpoint suppressor 1*) which is apparently involved in repressing expression of genes important for tumorigenesis [34]. *CHES1 *mRNA has been reported as down-regulated in oral squamous cell carcinoma [35] and in hepatocellular carcinoma [36]. Here we found *CHES1 *mRNA levels to be significantly (-2.03 fold; P = 0.045) [expression values 920.32 vs. 452.95], down-regulated in BCC compared to normal skin. Not unexpectedly, mRNAs encoding proteins involved in inducing apoptosis were also found to be down-regulated. These include *CIDE *[37] and *CARD15 *[38] which are 4.18 fold (P = 0.029) [expression values 415.5 vs. 99.41] and 2.31 fold (P = 0.031) [expression values 313.08 vs. 135.42], down-regulated in BCC compared to normal skin.

Using support vector machine analysis we have identified 6 transcripts that, as a group, enable the accurate classification of all 25 specimens as BCC or normal skin. These include *matrix metalloproteinase 11/mmp11 *(previously associated with the presence of other cancer types, including oral [39,40], lung [41] and breast [42]); *hydroxysteroid (17-beta) dehydrogenase 4/hsd17b4 *(changes in expression of which have been associated with breast cancer ([43]); *B-cell receptor-associated protein 29/bap29 *(a member of the B cell receptor-associated family of proteins [44-46]); *1-acylglycerol-3-phosphate O-acyltransferase 3/agpat3 *(which catalyses the acylation of lysophosphatidic acid to form phosphatidic acid, the precursor of all glycerolipids [47]); as well as *zinc finger protein 548 *and *full-length cDNA clone CS0DK008YI09 of HeLa cells Cot 25-normalized of homo sapiens*, on which no literature has previously been published. Future studies involving the co-analysis of this group of 6 transcripts in larger cohort of BCC and normal skin specimens should enable validation of their diagnostic relevance.

Molecular events responsible for the quite unique invasive, but non-metastatic, nature of BCCs are not known. However, it is interesting to note that expression of certain genes believed to be involved in malignant invasion and metastasis of another form of skin cancer, *i.e*. malignant melanoma, apparently differ in their expression patterns in BCCs. *AP-2 *transcription factor is not expressed in malignant melanoma cells [48], but it is significantly up-regulated (by 10.7 fold) [expression values 227.05 vs. 2,423.26], in BCC specimens compared to normal skin. Conversely, increased expression of *EGF-R *is associated with melanomas metastasis [48], but its expression is down-regulated (by approximately 1.2 fold) [expression values 76.91 vs. 62.78], in BCCs. Unlike BCCs, breast cancers frequently metastasise. Interestingly, in our microarray study of 104 breast tumours and normal breast tissue (*manuscript in preparation*) we identified changes in expression patterns of syndecan adhesion receptors (*for review*: see [49]) *i.e*. *syndecan 1 *is up-regulated, and *syndecan 2 *is down-regulated, in breast tumours compared to normal breast tissue. In this study of non-metastatic BCCs, we found *syndecan 1 *to be approximately 1.6 fold down-regulated [expression values 3,488.91 vs. 2,124.43], and *syndecan 2 *to be approximately 3 fold [expression values 160.21 vs. 559.84] up-regulated compared to levels in normal skin tissue. Furthermore, ankyrin (encoding membrane-associated cytoskeletal proteins) binding to membrane molecules has been suggested as necessary for cell adhesion, migration and tumour metastasis [50]. In our breast cancer study we found *ankyrin 3 *expression levels to be up-regulated compared to normal tissue, while here we report *ankyrin 3 *levels to be down-regulated (approximately 1.6 fold) [expression values 350.02 vs. 224.48], in BCC compared to normal tissue. While functional studies would be required to determine a causative/direct, rather than an associative, role for transcripts such as *AP-2*, *EGF-R*, *syndecan 1 *&*2*, and *ankyrin 3 *in controlling metastasis, the results from this study suggest that expression of the mRNAs may be, in some way, involved in this process.

Differential expression, between BCC and normal skin, of many transcripts not previous associated with the presence of BCC was also found during the course of our study. Basonuclin (now termed Basonuclin 1; was first discovered in cultured human epidermal cells [51]) and the more recently discovered basonuclin 2 [52] are zinc finger proteins. Basonuclin 1 is expressed at high levels in proliferating keratinocytes of stratified squamous epithelium. During terminal differentiation of squamous epithelium, *basonuclin *mRNA and protein disappear from the suprabasal cells [53,54]. In normal cultured human keratinocytes, *basonuclin 1 *is the predominant transcript – with expression levels approximately 10 fold that of *basonuclin 2 *[52]. In this study we found that *basonuclin 1 *levels did not differ significantly between BCC and normal skin, although a previous study of 3 BCC and 2 normal skin specimens indicated increased expression of *basonuclin 1 *in BCC, dependent on Gli protein expression [55]. In contrast, we report *basonuclin 2 *levels to be significantly increased in BCCs. This finding was observed with all 4 *basonuclin 2 *probe sets present on the microarray, indicating a 6.7–9.6 fold increase level (P < 0.00005) of expression [greatest change in expression values being 85.63 vs. 828.01], in BCC. This increased expression of *basonuclin 2 *in BCC compared to normal skin was confirmed by qPCR analysis. This, we believe, is the first study indicating an association between expression of *basonuclin 2 *and BCC.

ABCC12/mrp9, identified in 2001 [56] is one of a super-family of 9 ATP-binding cassette (ABC) multiple drug resistant proteins [57]. *Mrp9 *encodes an approximately 100 kDa protein detectable in breast cancer, normal breast tissue and testis, while an alternative *mrp9 *transcript – encoding an approximately 25 kDa protein – has been detected in normal brain, skeletal muscle and ovary tissues. Due to the differential levels of expression of *mrp9 *transcripts in breast tumour and normal tissue, MRP9 has been proposed as an immunotherapy target for breast cancer [58]. The functional relevance of our observation of approximately 8.7 fold greater levels of *mrp9 *[expression values 21.12 vs. 184.71] in BCC compared to normal skin has yet to be determined; its presence is unlikely to be involvement in drug resistance, as all of the BCCs included in this study were chemotherapy-naive (indeed, except in a limited number of advanced cases of BCC, chemotherapy is not used as a therapy for this disease. In these exceptional cases, excellent response rates have been reported with cisplatin in combination with either 5-fluorouracil or doxorubicin [59]). However, as for breast cancer, *mrp9 *mRNA may be useful as a member of a panel of BCC biomarkers or as an immunotherapy.

Chromagranin A (ChgA; parathyroid secretory protein 1) is an established tissue marker associated with neuroendocrine differentiation – and indicative of outcome – in non-small cell lung carcinoma [60]. Increased levels of ChgA in serum have been associated with poor prognosis/shortened survival for prostate cancer patients [61]. ChgA protein levels have been proposed to assist in the diagnosis of Merkel cell carcinoma patients who may benefit from oncological therapy [62-64]. Although described as relatively uncommon – using analysis of markers including ChgA – neuroendocrine differentiation in BCC has been reported [65]. In this study we have found *ChgA *levels to be significantly (130.3 fold; P = 0.000001) [expression values 19.31 vs. 2,516.41], up-regulated in BCCs compared to extremely low levels in normal skin specimens. This is in agreement with the observation of ChgA protein detectable in 55% (11/20) BCC specimens [66]. By qPCR analysis, *ChgA *was undetected in normal skin but was present in BCC specimens. Interestingly, other neuroendocrine markers, including *SNAP-25 *(3.24 fold; P = 0.008) [expression values 40.17 vs. 130.25] and *neuroendocrine protein 1/7B2 protein *(3.48 fold; P = 0.0001) [expression values 46.38 vs. 161.44], were also increased in the BCC specimens. These results indicate *ChgA *to be a potentially very useful marker for BCC.

In this study we present the first whole genome expression microarray analysis of skin cancer, aimed at investigating the molecular profile of BCC in comparison to non-cancerous skin biopsies. This investigation has not only confirmed previous findings from analyses of limited numbers of transcripts, but it has also identified changes in expression of mRNAs that had never previously been associated with this disease. The results from this work are interesting and exciting, but it is necessary to recognise their preliminary nature. Further analyses, building on our findings, should include independent replication studies so that the true relevance of these findings may be realised.

## Conclusion

The success of this study indicates the feasibility and relevance of using whole genome microarrays to study BCC specimens. In addition to confirming previous findings, this work has increased our understanding of molecular differences between BCC and normal skin and has identified a number of novel potential biomarkers for BCC. Future studies including BCC tissue and normal skin tissue from the same individual, thus lowering inter-individual variability and ruling out genetic influences; analyses of age- and gender-matched cases; studies focusing on molecular profiling and comparisons of sub-types of BCC, with due consideration given to disease duration (as early tumours may have a different gene expression profile to prolonged tumours); as well as analysing BCCs that metastasise compared to those that do not, should further increase our understanding of this disease and assist in management of the individual BCC patients. Furthermore, as gene expression may be independent of protein levels, future confirmatory analysis at the protein level would complement these findings.

## Materials

### Patient Characteristics

This study involved analysis of 20 BCC biopsies from both male and female patients aged between 47 years and 83 years (mean age = 65 +/- 11 years; median = 67 years) at the time of diagnosis. In order to gain an understanding of the most common types of BCC, we elected to include a range of BCC sub-types in this study, rather than to focus on any particular sub-type. For this reason, BCC sub-classifications included were nodular/micronodular, superficial and sclerosing. Tissue specimens from these twenty cases of BCC were procured at Blackrock Clinic and the Bons Secours Hospital, Dublin, examined macroscopically, immediately snap-frozen in liquid nitrogen, and were subsequently stored at -80°C until required for analysis. Five normal skin specimens (from consenting male and female volunteers of a similar age range who do not/never had skin cancer) were also included in these studies.

### RNA Extraction

For RNA analyses, dissected tumours that had been snap-frozen in liquid nitrogen and then stored at -80°C until required were homogenised, on ice, in 1 ml TriReagent (Sigma; Poole, England) and total RNA was subsequently isolated according to the manufacturer's instructions. RNA quantity and purity were assessed at 260 nm and 280 nm using a Nanodrop (ND-1000; Labtech. International); an Agilent bioanalyser (Agilent 2100; Agilent Technologies) was used to assess RNA qualitatively after isolation and, subsequently, after biotin-labelling and after fragmentation.

100 ng of each specimen was amplified and labelled using the Affymetrix GeneChip Eukaryotic 2 Cycle Labelling Assays for Expression Analysis, (Affymetrix; 900494) according to the manufacturer's instructions . Gene expression was examined using whole genome microarrays (Affymetrix; U133 Plus 2.0; 900470).

### Microarray hybridization

Hybridisation solution (1 mol/l NaCl, 20 mmol/1 EDTA, 100 mmol/1 2-(N-morpholino) ethanesulfonic acid, and 0.01% Tween 20) was used to pre-hybridise Affymetrix; U133 Plus 2.0 oligonucleotide microarrays for 10 minutes at 45°C and 60 rpm. The pre-hybridisation solution was removed and replaced with 200 μl hybridisation solution containing 0.05 μg/μl fragmented cRNA. The arrays were hybridised for 16 hours at 45°C and 60 rpm. Arrays were subsequently washed (Affymetrix Fluidics Station 400) and stained with streptavidin-phycoerythrin (Stain Buffer, 2 mg/ml acetylated BSA and 10 μg/ml streptavidin R-phycoerythrin; Molecular Probes, Inc., Eugene, OR), and were scanned on an Affymetrix GCS GeneChip GeneArray scanner. Resulting data was analysed using GCOS, dCHIP, and GeneSpring (Agilent Technologies).

### Normalisation and Filtering

Cel files obtained from the GCOS server were processed and normalized by dCHIP [67] algorithm. In this normalisation procedure, an array with median overall intensity is chosen as the baseline array against which other arrays are normalized at probe intensity level. Subsequently, a subset of PM probes, with small within-subset rank difference in the two arrays, serves as the basis for fitting a normalization curve. A filter was designed to include a fold change of at least 1.2 fold between normal and BCC specimens, a difference of at least 100 Affymetrix arbitrary units between normal and BCC average values, and a t-test between normal and BCC (with a p-value cut-off <0.05).

### Gene Ontology and Pathway Analysis Analysis

In order to establish which gene ontologies (GO) are over-represented in our lists of 2,108 significantly up-regulated and 1,813 significantly down-regulated (in BCCs compared to normal skin) transcripts, we compared these to the list of all human genes from the EBI [68], using Gostat [69]. In brief, for all of the gene transcripts analysed, GOstat determines the associated annotated GO terms and all branches/splits on their connection path. The program then counts the number of appearances of each GO term for the gene transcripts in the list being analysed, as well as in the reference list. For each GO term, a Chi-squared p-value is calculated representing the probability that the observed numbers of counts could have resulted from randomly distributing this GO term between the tested and the reference lists. If the expected value for any analysis is <5, the Chi-squared approximation is considered to be inaccurate. Genmapp [70] was used to identify pathways affected by the differentially-expressed genelist.

### Real-time PCR (qPCR)

Following priming with oligo (dT) at 65°C for 5 minutes, followed by 1 minute incubation on ice, cDNA was synthesised from 100 ng total RNA, using Superscript III RNase H- (with increased thermal stability; Invitrogen), RNase OUT Ribonuclease (active against RNase A, B and C; Invitrogen) and a cocktail of dNTPs, by incubating at 50°C for 1 hour, followed by 70°C for 15 minutes, in a 40 μl reaction volume. The cDNA (diluted 1:10), was amplified in 25 μl reactions, by qPCR, using an ABI 7500 Real-Time PCR System (Applied Biosystems, Foster City, CA). Following evaluation of 11 potential endogenous controls in a random selection* of 4 BCC and 4 normal specimens, this study involved evaluation, in all 20 BCC and 5 normal skin specimens, of 5 target transcripts normalised to 2 suitable endogenous controls – β-actin and GAPDH – and calibrated against a pooled cDNA of BCC and normal skin specimens, the relative quantity of which was set to 1. The temperature profile of all reactions was 50°C for 2 minutes 95°C for 10 minutes, 40 cycles of 95°C and 60°C for 1 minute. Individual specimens were analysed in triplicate. [* *Note*: these 4 BCC and normal specimens were analysed by microarrays with all of the other specimens included in this study].

## Abbreviations

BCC – basal cell carcinoma; qPCR – quantitative-polymerase chain reaction

## Competing interests

The author(s) declare that they have no competing interests.

## Authors' contributions

LOD participated in the design and co-ordination of the study, secured financial support for this research, was involved in RNA isolation & study by microarrays and qPCR, data analysis and interpretation, and she drafted the manuscript; JMM participated in RNA isolations, quality assessment, and in preparation of specimens for microarray analysis; PD participated in analysing labeled specimens on microarrays chips and in bioinformatics analysis; EMK performed qPCR analysis; JPM and ER were involved in the bioinformatics analysis; PG and HJ were involved in analysing labeled specimens on microarrays chips; NOD was involved in RNA isolation; NW provided the clinical specimens/anonymised clinical data and was involved in raising financial support for this research; MC participated in the design of the study, and contributed to data interpretation, drafting of the manuscript and he was involved in raising financial support for this research. All authors approved the final manuscript.

## Supplementary Material

Additional File 1 Table 2 [Additional File 1]. Gene transcripts significantly up-regulated in BCC compared to normal skin specimens Click here for file

Additional File 2 Table 3 [Additional File 2]. Gene transcripts significantly down-regulated in BCC compared to normal skin specimens Click here for file

Additional File 3 Figure 3 [Additional File 3]. qPCR analysis of 10 transcripts in four BCC and four normal specimens as potential endogenous controls for analysis involving validation of microarray data in all specimens. Click here for file
