# Supplementary figures and images for: Investigation of the molecular profile of basal cell carcinoma using whole genome microarrays
Source: Mol Cancer. 2006 Dec 15;5:74. doi: 10.1186/1476-4598-5-74 (PMC1770933; doi:10.1186/1476-4598-5-74)

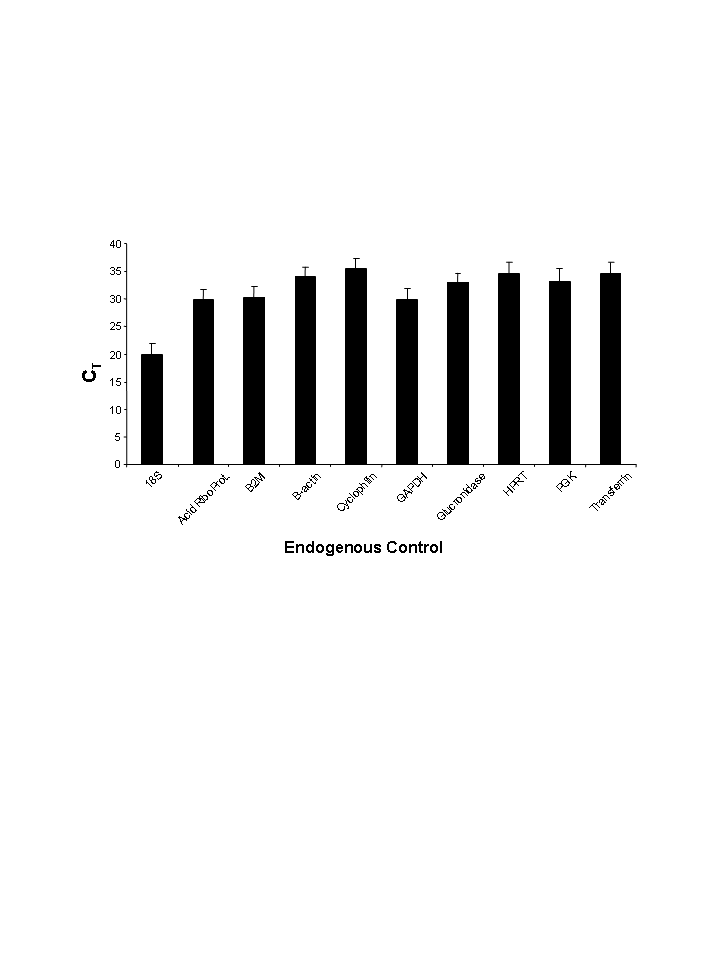

Supplement: Additional File 3 — Figure 3 [Additional File 3]. qPCR analysis of 10 transcripts in four BCC and four normal specimens as potential endogenous controls for analysis involving validation of microarray data in all specimens. [file 1476-4598-5-74-S3.tiff]
